# A new mapping method for quantitative trait loci of silkworm

**DOI:** 10.1186/1471-2156-12-19

**Published:** 2011-01-28

**Authors:** Hai-Ming Xu, Chang-Shuai Wei, Yun-Ting Tang, Zhi-Hong Zhu, Yang-Fu Sima, Xiang-Yang Lou

**Affiliations:** 1 Institute of Bioinformatics, College of Agriculture and Biotechnology, Zhejiang University, Hangzhou 310029, China; 2 Ningbo Institute of Technology, Zhejiang University, Ningbo 315100, China; 3 College of Agriculture Science and Technology, Soochow University, Suzhou 215006, China; 4 Department of Biostatistics, School of Public Health, University of Alabama at Birmingham, Birmingham, Alabama 35294, USA

## Abstract

**Background:**

Silkworm is the basis of sericultural industry and the model organism in insect genetics study. Mapping quantitative trait loci (QTLs) underlying economically important traits of silkworm is of high significance for promoting the silkworm molecular breeding and advancing our knowledge on genetic architecture of the Lepidoptera. Yet, the currently used mapping methods are not well suitable for silkworm, because of ignoring the recombination difference in meiosis between two sexes.

**Results:**

A mixed linear model including QTL main effects, epistatic effects, and QTL × sex interaction effects was proposed for mapping QTLs in an F_2 _population of silkworm. The number and positions of QTLs were determined by *F*-test and model selection. The Markov chain Monte Carlo (MCMC) algorithm was employed to estimate and test genetic effects of QTLs and QTL × sex interaction effects. The effectiveness of the model and statistical method was validated by a series of simulations. The results indicate that when markers are distributed sparsely on chromosomes, our method will substantially improve estimation accuracy as compared to the normal chiasmate F_2 _model. We also found that a sample size of hundreds was sufficiently large to unbiasedly estimate all the four types of epistases (i.e., additive-additive, additive-dominance, dominance-additive, and dominance-dominance) when the paired QTLs reside on different chromosomes in silkworm.

**Conclusion:**

The proposed method could accurately estimate not only the additive, dominance and digenic epistatic effects but also their interaction effects with sex, correcting the potential bias and precision loss in the current QTL mapping practice of silkworm and thus representing an important addition to the arsenal of QTL mapping tools.

## Background

Silkworm (*Bombyx mori*) is the basis of sericultural industry. With nearly 5000 years' domestication, silkworm has an undoubted importance in human history and is still of great value in modern economy. In addition, it is also an ideal model organism of the Lepidoptera. Because silkworm is easy to rear and could produce large amount of mutation, it is second to fruit fly as a model organism in insect genetics study. Over these years, the "old" creature is becoming a new hot spot in genetic research.

Many important traits of silkworm are complex quantitative traits, such as whole cocoon weight and cocoon shell weight, etc. The genetic variation of quantitative traits are usually controlled by a number of genes (quantitative trait loci, QTLs) with epistatic and gene × environment interactions. To locate their positions on chromosome and estimate their contribution to the variation of trait is a key step for positional cloning and follow-up utilization of those genes. With the development of modern molecular biology, it has become possible to dissect the genetic mechanism of quantitative trait and to identify the associated genes and their interacting network by co-segregation analysis of molecular markers in a mapping population based on specific genetic model connecting QTL genotype with a phenotype of interest.

Since the draft sequence of silkworm genome [1] was reported, genetic research of silkworm has been greatly spurred and many linkage-maps have been constructed with various molecular makers, such as random amplified polymorphic DNAs (RAPDs), amplified fragment length polymorphisms (AFLPs), selective amplification of DNA fragments (SADFs), microsatellites (also known as simple sequence repeats, SSRs) and expressed sequence tags (ESTs) [2-6]. Meanwhile, many statistical models were developed for mapping QTLs, such as the interval mapping (IM) method [7] and the composite interval mapping (CIM) method [8]. Along with increasing evidence supporting the claim that epistatic and QTL-environment interactions are usually involved in the genetic variation of complex trait [9-11], several complicated mapping models were developed to analyze epistatic effects [12-18]. Kao et al. [12] expanded CIM to multiple interval mapping (MIM) for detecting epistasis. Wang et al. [15] established a mixed linear model based composite interval mapping (MCIM) method to analyze both epistasis and *QE *interaction in a double haploid (DH) population. A few years later, the MCIM was extended for other designed populations [19] and improved in searching strategy and estimating QTL parameters [16]. Parallel to these frequentist methods, Bayesian approaches have also been proposed for QTL mapping of complex traits [17,20-24]. Recently, there are several new Bayesian methods developed for mapping QTLs underlying dynamic traits [25], ordinal traits [26], multiple traits [27-30], expression data [31], and gene frequency data [32], and for multiple inbreed lines designs [33].

Although there are a large number of QTLs reported in other species, relatively fewer QTL mapping studies are performed in silkworm [6,34-37], which in part result from the fact that the current models and corresponding analysis methods are not appropriate for the silkworm. Silkworm has a particular characteristic called female achiasmata where meiosis occurs with no crossover between homologous chromosome pairs. Yet the majority of recently developed mapping tools are based on the assumption of chiasmata without considering the genetic differences between male and female. Therefore, only specific mapping populations such as a backcross (BC) population are suggested for gene mapping in order to satisfy this assumption [3,5]. By setting female silkworm as the homozygous parental lines, they can avoid the problem of achiasmata. However, a BC population contains fewer segregant types of molecular markers than F_2 _population. As a result, genetic information is not enough to reveal additive and dominance effects, epistatic effects of QTLs and their interaction effects with environment. Therefore, it is necessary to develop a new method of QTL mapping with consideration of female achiasmata for F_2 _population of silkworm.

In the present study, we proposed a new statistical method for QTL mapping in silkworm. The method can analyze the additive, dominance and digenic epistic effects of QTLs, as well as their interaction with sex. The effectiveness of the method is investigated by intensive Monte Carlo simulations.

## Methods

### Genetic model for QTL mapping of silkworm

To specify the achiasmate characteristic of silkworm, an F_2 _population is used to illustrate our methods. Models for other mapping populations or experimental designs can be established by including or excluding relevant QTL main effects or QTL × environment interaction effects. Without loss of generality, we assume here that the phenotypic value of a quantitative trait in a F_2 _population is controlled by additive and dominant effects of *n *QTLs and *m *digenic epistatic effects of QTLs. Because there are differences in both the genetic material and meiosis mode between male and female silkworms, sex effects should be involved in the genetic variation of silkworm traits (e.g. cocoon quality trait). As sex functions as an endogenous environment for the development of an autosomal sex-specific trait, sex is treated as a pseudo environmental covariate in our experimental design. When necessary, the interaction effects between QTLs and sex can be included in our model. Therefore, the phenotypic value of individual *i *with sex *h *(*i *= 1, 2,..., *t*_*h*_; *h *= 1, 2) can be expressed as the following mixed linear model which is extended from those in Gao and Zhu [19] and in Yang et al. [16]:

(1) $$\begin{array}{c} y_{hi}=\mu +\sum_{k=1}^{n}\left(x_{Aik}a_{k}+x_{Dik}d_{k}\right)+\sum_{l=1}^{m}(x_{AAil}aa_{l}+x_{ADil}ad_{l}+x_{DAil}da_{l}+x_{DDil}dd_{l})\\ +s_{h}+\sum_{k=1}^{n}(x_{Ahik}as_{hk}+x_{Dhik}ds_{hk})\\ +\sum_{l=1}^{m}\left(x_{AAhil}aas_{hl}+x_{ADhil}ads_{hl}+x_{DAhil}das_{hl}+x_{DDhil}dds_{hl}\right)+\varepsilon_{hi}, \end{array}$$

where μ is the population mean; *a*_*k *_and *d*_*k *_are the additive effect and dominant effect of the *k*-th QTL, respectively; *x*_*Aik *_and *x*_*Dik *_are the coefficients of QTL effects which can, when QTL genotypes are unobserved, be derived from the conditional probability of the putative QTL given the genotypes of flanking markers (flanking marker *M*_+_, *M*_- _of QTL *Q*) and the QTL position (the recombination frequency *r*_*M+Q*_,*r*_*M-Q*_), respectively; *aa*_*i*_, *ad*_*i*_, *da*_*i *_and *dd*_*i *_are the additive-additive, additive-dominance, dominance-additive and dominance-dominance epistatic effects of the *l*-th pair of QTLs, respectively, with their coefficients *x*_*AAil*_, *x*_*ADil*_, *x*_*ADil *_and *x*_*DDil*_, which are the products of the corresponding *x*_*A *_and *x*_*D*_; all the above additive, dominance and epistatic effects are of our interest and thus considered as fixed; *S*_*h *_is the sex effect of sex *h*, *S*_*h *_~ (0, σ^*2*^_*S*_); *as*_*hk *_and *ds*_*hk *_are additive-sex and dominance-sex interaction effects, *as*_*hk *_~ (0, σ^*2*^_*AS*_) and *ds*_*hk *_~ (0, σ^*2*^_*DS*_), respectively; *aas*_*hl*_, *ads*_*hl*_, *das*_*hl *_and *dds*_*hl *_are interaction effects between epistasis and sex, *aas*_*hl *_~ (0, σ^*2*^_*AAS*_), *ads*_*hl *_~ (0, σ^2^_*ADS*_), *das*_*hl *_~ (0, σ^2^_*DAS*_) and *dds*_*hl *_~ (0, σ^2^_*DDS*_); ε_*hi *_is the random residual effect, ε_*hi *_~ (0, σ^*2*^_*e*_).

The above model can be expressed in the matrix form:

(2) $$\begin{array}{l} y=1\mu +X_{A}b_{A}+X_{D}b_{D}+X_{AA}b_{AA}+X_{AD}b_{AD}+X_{DA}b_{DA}+X_{DD}b_{DD}\text{+}U_{S}e_{S}\\ +U_{AS}e_{AS}+U_{DS}e_{DS}+U_{AAS}e_{AAS}+U_{ADS}e_{ADS}+U_{DAS}e_{DAS}+U_{DDS}e_{DDS}+e_{\varepsilon }\\ \text{=}Xb+\sum_{u=1}^{r+1}U_{u}e_{u}\text{\textasciitilde{}}N(Xb,\;V=\sum_{u=1}^{r+1}\sigma_{u}^{2}U_{u}R_{u}U_{u}^{T}) \end{array}$$

where **y **is the vector of the phenotypic values; **b **is the vector of fixed effects and **X **is the coefficient matrix; **e**_*u *_is the vector of the *u*-th random effect and **U**_*u *_is the corresponding coefficient matrix, and **R**_*u *_is an identity matrix for every *u *in this model if the components of **e**_*u *_are independent.

Since the QTL genotype of each individual is unobservable in the real experiment, the coefficients of QTL effects are unknown. However, they could be substituted with their expectation based on the conditional probability of QTL genotype given the genotypes of flanking markers. The expected coefficients of additive and dominance effects given the flanking markers are listed in Table 1, while the coefficients of epistatic effects and QTL × sex interaction effects can be calculated by multiplying the corresponding *x*_*A*_, *x*_*D*_, and coefficients of sex effects (0 or 1). When the information on a portion of markers is missing, the algorithm based on transitional possibility matrix, proposed by Jiang and Zeng [38], can be applied to impute missing data. As shown in Table 1, the major difference between our achiasmate model and the traditional chiasmate model lies in calculation of the expected coefficients.

**Table 1:** Conditional probabilities of QTL genotypes and coefficients of additive and dominance effects for achiasmate and chiasmate F_2 _populations

Marker genotype	**Achiasmate F**_**2 **_**population **^**2**^					**Chiasmate F**_**2 **_**population **^**3**^		
	
	***QQ ***^**1**^	*Qq*	*qq*	***x***_***A***_	***x***_***D***_	*QQ*	*Qq*	*qq*
*M*_+_*M*_+_*M*_-_*M*_-_	*s*_1_*s*_2_/*s*	*r*_1_*r*_2_/*s*	0	*s*_1_*s*_2_/*s*	(*r*_1_*r*_*2 *_*- s*_1_*s*_2_)/2*s*	*s*_*1*_^2^*s*^2^_2_/*s*^2^	2*s*_1_*s*_2_*r*_1_*r*_2_/*s*^2^	*r*_*1*_^2^*r*_*2*_^2^/*s*^*2*^
*M*_+_*M*_+_*M*_-_*m*_-_	*s*_1_*r*_2_/*r*	*r*_1_*r*_2_/*r*	0	*s*_1_*r*_2_/*r*	(*r*_1_*s*_*2 *_*- s*_1_*r*_2_)/2*r*	*s*_1_*s*_2_*s*_1_*r*_2_/*sr*	(*s*_1_*s*_2_*r*_1_*s*_2 _+ *r*_1_*r*_2_*s*_1_*r*_2_)/*sr*	*r*_1_*r*_2_*r*_1_*s*_2_/*sr*
*M*_+_*M*_+_*m*_-_*m*_-_	-	-	-	-	-	*s*_*1*_^2^*r*^2^_2_/*r*^2^	2*r*_1_*s*_1_*r*_2_*s*_2/_*r*^2^	*r*_1_^2^*s*_2_^2^/*r*^2^
*M*_+_*m*_+_*M*_-_*M*_-_	*r*_1_*s*_2_/*r*	*s*_1_*r*_2_/*r*	0	*r*_1_*s*_2_/*r*	(*s*_1_*r*_*2 *_*- r*_1_*s*_2_)/2*r*	*s*_1_*s*_2_*r*_1_*s*_2_/*sr*	(*s*_1_*s*_2_*s*_1_*r*_2 _+ *r*_1_*r*_2_*r*_1_*s*_2_)/*sr*	*r*_1_*r*_2_*s*_1_*r*_2_/*sr*
*M*_+_*m*_+_*M*_-_*m*_-_	*r*_1_*r*_2_/2*s*	*s*_1_*s*_2_/*s*	*r*_1_*r*_2_/2*s*	0	(*s*_1_*s*_*2 *_*- r*_1_*r*_2_)/2*s*	$\frac{2s_{1}s_{2}r_{1}r_{2}}{s^{2}+r^{2}}$	$\frac{s_{1}^{2}s_{2}^{2}+r_{1}^{2}r_{2}^{2}+s_{1}^{2}r_{2}^{2}+r_{1}^{2}s_{2}^{2}}{s^{2}+r^{2}}$	$\frac{2s_{1}s_{2}r_{1}r_{2}}{s^{2}+r^{2}}$
*M*_+_*m*_+_*m*_-_*m*_-_	0	*s*_1_*r*_2_/*r*	*r*_1_*s*_2_/*r*	*-r*_1_*s*_2_/*r*	(*s*_1_*r*_*2 *_*- r*_1_*s*_2_)/2*r*	*r*_1_*r*_2_*s*_1_*r*_2_/*sr*	(*s*_1_*s*_2_*s*_1_*r*_2 _+ *r*_1_*r*_2_*r*_1_*s*_2_)/*sr*	*s*_1_*s*_2_*r*_1_*s*_2_/*sr*
*m*_+_*m*_+_*M*_-_*M*_-_	-	-	-	-	-	*r*_*1*_^*2*^*s*^2^_2_/*r*^2^	2*r*_1_*s*_1_*r*_2_*s*_2_/*r*^2^	*s*_*1*_^2^*r*^2^_2_*r*^2^
*m*_+_*m*_+_*M*_-_*m*_-_	0	*r*_1_*s*_2_/*r*	*s*_1_*r*_2_/*r*	-*s*_1_*r*_2_/*r*	(*r*_1_*s*_2 _- *s*_1_*r*_2_)/2*r*	*r*_1_*r*_2_*r*_1_*s*_2_/*sr*	(*s*_1_*s*_2_*r*_1_*s*_2 _+ *r*_1_*r*_2_*s*_1_*r*_2_)/*sr*	*s*_1_*s*_2_*s*_1_*r*_2_/*sr*
*m*_+_*m*_+_*m*_-_*m*_-_	0	*r*_*1*_*r*_2_/*s*	*s*_1_*s*_2_/*s*	-*s*_1_*s*_2_/*s*	(*r*_1_*r*_2 _- *s*_1_*s*_2_)/2*s*	*r*_*1*_^2^*r*_*2*_^2^/*s*^2^	2*s*_1_*s*_2_*r*_1_*r*_2_/*s*^2^	*s*_*1*_^2^*s*_*2*_^2/^*s*^2^

^1 ^*r*(=*r*_*M+M-*_), *r*_1_(=*r*_*M+Q*_), *r*_2_(=*r*_*M-Q*_) are recombination rates between marker *M*_+ _and marker *M*_-_, between *M*_+ _and QTL, between *M*_- _and QTL, respectively, under the assumption that the order of flanking markers and QTL is *M*_+_→*Q*→*M*_-_; *s *= 1*-r*, *s*_1 _= 1*-r*_1_, *s*_2 _= 1-*r*_2_.

^2 ^There are no marker genotypes of *M*_*+*_*M*_*+*_*m*_-_*m*_- _and *m*_*+*_*m*_*+*_*M*_-_*M*_- _in the achiasmate F_2 _population, and thus the corresponding conditional probability of QTL genotype is not provided.

^3 ^Double recombination is considered in calculation of conditional probability for chiasmate F_2 _popultion.

The above QTL full model, assuming the number of QTLs and their positions are known, can be used to detect the significance of QTL effects. Based on the final QTL full model after excluding insignificant QTLs, all genetic effects of QTL and QTL × sex interaction effects will be estimated by the mixed linear model approach.

### Scanning for QTLs with main effects

To remedy a potential bias in both the estimated effect values and position arisen from linked QTLs and control the residual background variation, Zeng [8] developed CIM method by integrating IM with multiple regression. Zeng [8] showed that, conditional on an intermediate marker, its two flanking markers would be independent of each other in a backcross population (it also holds true in a double haploid population), assuming no crossover interference, providing the theoretical basis of Zeng's separation of multiple linked QTLs. To avoid "ghost" QTL due to the impact of linked QTLs and control the background genetic variance, as in Zeng [8], we like first to select significant markers as background markers prior to genome-wide scanning for QTLs underlying silkworm traits. Pairs of adjacent markers are selected [39] and their effects are tested in the following model:

(3) $$y_{hi}=\mu_{h}+\zeta_{Ait}^{+}a_{th}^{+}+\zeta_{Dit}^{+}d_{th}^{+}+\zeta_{Ait}^{-}a_{th}^{-}+\zeta_{Dit}^{-}d_{th}^{-}+\varepsilon_{hi},$$

where *a*^+^_th _(*a*^-^_*th*_) and *d*^+^_*th*_(*d*^-^_*th*_) are the additive and dominance effects due to the right (left) marker of the *t*-th marker interval in the *h*-th sex, with corresponding coefficients ζ^+^_A_(ζ^-^_A_) and ζ^+^_D_(ζ^-^_D_); the other parameters have the same definition as those in model (1). ζ^+^_A_(ζ^-^_A_) takes value of 1, 0, -1 when the marker genotype is *MM*, *Mm *and *mm*, respectively. ζ^+^_D_(ζ^-^_D_) takes value of -0.5 for homozygous genotype (*MM *and *mm*) and 0.5 for heterozygote (*Mm*). If the marker genotype information is missing, the transitional-possibility-matrix algorithm will be employed to calculate their expected values. To determine which pair of adjacent markers should be selected, the *F*-testing is applied and the threshold value is determined by permutations [40]. After all the marker intervals exceeding the *F*-critical value are included into the model, stepwise model selection method is performed to eliminate all ghost peaks.

Once the marker intervals with significant effects are identified, genome-wide one-dimensional searching for QTLs can be conducted with inclusion of selected markers in the model to control the background effects from other unknown QTLs. The following model will be used to test a putative QTL *k*,

(4) $$y_{hi}=\mu_{h}+x_{Aik}a_{kh}+x_{Dik}d_{kh}+\sum_{t=1}^{mi}\left(\zeta_{Ait}^{+}a_{th}^{+}+\zeta_{Dit}^{+}d_{th}^{+}+\zeta_{Ait}^{-}a_{th}^{-}+\zeta_{Dit}^{-}d_{th}^{-}\right)+\varepsilon_{hi},$$

where *mi *represents the number of selected marker intervals; the other parameters are the same as defined in the models (1) and (3). The scanning is performed with a walk step, say 1 cM, within every selected marker interval. The significance of the putative QTL is tested by the following *F*-statistic:

(5) $$F=\frac{SSR(Q|M)/[rank(X_{QM})-rank(X_{M})]}{SSE/[n-rank(X_{M})]}$$

where *Q *denotes QTL genetic effects with coefficient matrix of **X**_*Q*_, and *M *denotes maker effects with coefficient matrix of **X**_*M*_, **X**_*QM *_is a matrix catenated by the **X**_*Q *_and **X**_*M*_; *n *is the number of observed values, *rank *(**X**_*QM*_) and *rank *(**X**_*M*_) are the ranks of matrix **X**_*QM *_and **X**_*M*_, respectively; *SSR*(*Q|M*) is the extra sum of squares due to the genetic effects of the putative QTL given the inclusion of *M *in the model; *SSE *is the residual sum of squares. *SSR*(*Q|M*) and *SSE *can be calculated using Henderson III method [41]. The permutation technique is used to determine the critical value of *F*-test. For all the QTLs detected to be significant at the level of 0.05, the stepwise selection is conducted to eliminate the false positive peaks.

### Scanning for paired QTLs with epistatic effects

In order to detect the paired QTLs with significant interaction effects, two-dimensional whole genome scanning strategy should be adopted, while, the QTL model also need to be extended to inclusion of epistatic effects of paired QTLs. Before scanning epistatic effects, we still perform marker selection procedure. All the possible marker interval pairs are tested in the following model:

(6) $$\begin{array}{c} y_{hi}=\mu_{h}+\zeta_{AAip}^{+}aa_{ph}^{+}+\zeta_{ADip}^{+}ad_{ph}^{+}+\zeta_{DAip}^{+}da_{ph}^{+}+\zeta_{DDip}^{+}dd_{ph}^{+}\\ +\zeta_{AAip}^{-}aa_{ph}^{-}+\zeta_{ADip}^{-}ad_{ph}^{-}+\zeta_{DAip}^{-}da_{ph}^{-}+\zeta_{DDip}^{-}dd_{ph}^{-}\\ +\sum_{t=1}^{mi}\left(\zeta_{Ait}^{+}a_{th}^{+}+\zeta_{Dit}^{+}d_{th}^{+}+\zeta_{Ait}^{-}a_{th}^{-}+\zeta_{Dit}^{-}d_{th}^{-}\right)+\varepsilon_{hi}, \end{array}$$

where *aa*^+^_*ph*_(*aa*^-^_*ph*_), *ad*^+^_*ph*_(*ad*^-^_*ph*_), *da*^+^_*ph*_(*da*^-^_*ph*_) and *dd*^+^_*ph*_(*dd*^-^_*ph*_) denote the additive-additive, additive-dominance, dominance-additive and dominance-dominance epistatic effects within the *h*-th sex between two right (left) markers of the *p*-th marker interval pairs, respectively; the coefficients of epistatic effects can be calculated by the products of coefficients of marker major effects in model (3); other parameters are defined the same as those in model (4). For each paired marker intervals tested, the *F*-statistic to test its extra effects is calculated using the formula (5), and the critical value to declare significance is specified by calculating *F*-statistic in a series of randomly shuffling observation vector **y **s. All paired intervals above the critical value are then picked up as significant candidate interactions.

Suppose there are *mp *pairs of marker intervals selected. Within two paired marker intervals, the epistatic effects from two paired putative QTLs is tested in two-dimensional searching strategy. For the *l*-th paired putative QTLs, the following mapping model can be analyzed with inclusion of the epistatic effects of the other selected marker intervals and the main effects of the QTLs detected in one-dimensional scanning,

(7) $$\begin{array}{c} y_{hi}=\mu_{h}+x_{AAil}aa_{lh}+x_{ADil}ad_{lh}+x_{DAil}da_{lh}+x_{DDil}dd_{lh}\\ +\sum_{p=1}^{mp}\left(\zeta_{AAip}^{+}aa_{ph}^{+}+\zeta_{ADip}^{+}ad_{ph}^{+}+\zeta_{DAip}^{+}da_{ph}^{+}+\zeta_{DDip}^{+}dd_{ph}^{+}\right)\\ +\sum_{p=1}^{mp}\left(\zeta_{AAip}^{-}aa_{ph}^{-}+\zeta_{ADip}^{-}ad_{ph}^{-}+\zeta_{DAip}^{-}da_{ph}^{-}+\zeta_{DDip}^{-}dd_{ph}^{-}\right)\\ +\sum_{k=1}^{mi}\left(x_{Aik}a_{kh}+x_{Dik}d_{kh}\right)+\varepsilon_{hi} \end{array}$$

where *mp *is the number of selected interval pairs, *mi *is the number of QTLs identified in one-dimensional searching; all other parameters are defined the same as those in model (1) and model (6). Similar *F*-test and selection procedure are applied.

### Estimation of QTL parameters in the full model

After the number and positions of QTLs are specified, a full model consisting of all genetic effects of QTLs and their interaction effects with environment (sex) is established. The variance components of random effects can be estimated by restricted maximum likelihood (REML), the fixed effects by generalized least squares (GLS) or ordinary least squares (OLS), and the random effects by adjusted unbiased prediction (AUP). These mixed-model estimates of QTL effects are set to be the initial values of MCMC methods [42]. The sample distributions of QTL parameters are obtained by the Gibbs sampling [16,43]. Finally, each effect is estimated by the distribution mean, while significance of an effect is tested by *t *statistic.

### Numerical calculation of the difference in the coefficients of QTL effects between the achiasmate and the chiasmate models

As there is no genetic material exchange for female silkworm when producing gamete in meiosis, the marker frequency distribution and the conditional probabilities of QTL genotypes in silkworm F_2 _population are substantially different from those in the normal F_2 _population with chiasma. To demonstrate the difference in QTL detection and investigate the inappropriateness of QTL mapping of silkworm traits based on the traditional genetic model, we compared the conditional probabilities of QTL genotypes given the flanking marker genotypes under the achiasmata F_2 _and the traditional chiasmata F_2 _models that were calculated from Table 1.

We used two cases where *r *equal to 0.09 (10 cM) and 0.16 (20 cM), respectively, to evaluate the difference in the additive and dominance coefficients between two models summarized in the following steps: (1) to set *r *fixed and *r*_1 _changed from 0 to *r*; (2) to calculate each coefficient in two models based on the QTL conditional probability in Table 1 for every given marker genotype; (3) to calculate the absolute difference (*D*_*i *_= |*x*_*ai *_- *x*_*ci*_|) and relative difference (*R*_*i *_= |*x*_*ai *_- *x*_*ci *_|/*x*_*ai*_) for the *i*-th flanking marker genotype (7 totally), *x*_*a *_(*x*_*c*_) is the coefficient of QTL effect in the achiasmate (chiasmate) model; and (4) to investigate the maximum and the minimum of the set of *D*_*i *_and *R*_*i *_(*i *= 1, 2,..., 7) for every *r*_1_.

### Simulation scenarios

To investigate the efficiency and accuracy of the proposed methods, 300 simulations were conducted with the following QTL configuration. 5 chromosomes and 7 QTLs were considered. Each chromosome had 11 molecular markers and 10 equal marker intervals of 10 cM; 7 QTLs (Q1, Q2, Q3, Q4, Q5, Q6, Q7) were scattered on 5 chromosomes (see Table 2 and Table 3 for details), wherein, three pairs of QTLs E1 (Q1-Q7), E2 (Q3-Q5), E3 (Q6-Q7) were involved in epistatic effects while no additive effects, dominance effects, additive-sex interaction effects or dominance-sex interaction effects were set for Q6 and Q7 (Tables 2 and 5). Detailed information about the positions and effects is presented in Tables 2, 3, 4 and 5. The simulations were performed based on an F_2 _population with 300 individuals in which the numbers of males and females are equal. The proportions of total variation due to genetic effects and genetic × environment interaction effects were 50.47% and 19.53%, respectively; the narrow heritability was 33.56%. Three QTLs (Q2, Q3, Q4) and all epistatic pairs were considered to interact with sex having a variance ranging from 4 to 6.24, while Q1 and Q5 had no interaction with sex.

**Table 2:** Estimation of QTL positions and main effects with Models I, II and III ^a^

**QTL **^**b**^	**Chr**.	**Pos**.				*A*				*D*				Power		
		
		**Par**.	Est.(*SD*)			**Par**.	Est.(*SD*)			**Par**.	Est.(*SD*)					
										
			I	II	III		I	II	III		I	II	III	I	II	III
Q1	1	44	44.02 (3.14)	44.07 (3.43)	44.02 (3.14)	3.88	3.88 (0.38)	3.92 (0.52)	3.91 (0.43)	-2.3	-2.21 (0.53)	-2.27 (0.73)	-2.22 (0.60)	96.7	93.3	96.7
Q2	2	45	45.20 (4.09)	45.36 (5.40)	45.20 (4.09)	-2.4	-2.43 (0.42)	-2.48 (0.54)	-2.48 (0.48)	1.9	1.80 (0.51)	1.83 (0.76)	1.84 (0.61)	91	84.3	91
Q3	3	50	50.18 (2.13)	50.21 (3.27)	50.18 (2.13)	3.2	3.25 (0.38)	3.29 (0.48)	3.33 (0.44)	2.1	2.05 (0.52)	2.13 (0.74)	2.09 (0.61)	98.3	94.7	98.3
Q4	4	73	73.24 (4.23)	73.29 (5.77)	73.24 (4.23)	-2.8	-2.67 (0.41)	-2.74 (0.50)	-2.71 (0.44)	-1.9	-1.84 (0.53)	-1.87 (0.73)	-1.85 (0.60)	92	87.7	92
Q5	5	15	15.28 (6.40)	16.52 (7.94)	15.28 (6.40)	1.9	1.98 (0.36)	2.17 (0.52)	2.13 (0.42)	0	-0.01 (0.55)	-0.09 (0.85)	-0.08 (0.67)	62.7	49.7	62.7
Q6	2	75	-	-	-	0	-	-	-	0	-	-	-	-	-	-
Q7	4	24	-	-	-	0	-	-	-	0	-	-	-	-	-	-

^a ^Pos. (position): the distance (cM) between the QTL and the first marker on the same chromosome; *A*: additive effect; *D*: dominance effect; Chr. (chromosome): the chromosome on which the QTL is located; Power: the percentage of the QTL that is detected correctly to be significant at 0.05 and within the 95% confidence interval; Par. (parameter): the true parameter value in simulations; Est.: the estimate of parameter; *SD*: the standard deviation of the estimate from the true value that is listed in the parenthesis where applicable; I (Model I): model (1) (silkworm F_2 _model); II (Model II): the normal chiasmate F_2 _model; III(Model III): the reduced version of model (1) without all epistatic effects and their interaction effects with sex.

^b ^Q6 and Q7 are two QTLs without main effects, and thus could not been detected by the model of QTL main effects.

**Table 3:** Estimation of the positions and epistatic effects of the paired QTLs with Models I and II ^a^

**Epi**.	Pos. *i*			Pos*. j*			*AA*			*AD*			*DA*			*DD*			Power	
	
	**Par**.	Est.(*SD*)		**Par**.	Est.(*SD*)		**Par**.	Est.(*SD*)		**Par**.	Est.(*SD*)		**Par**.	Est.(*SD*)		**Par**.	Est.(*SD*)		I	II
														
		I	II		I	II		I	II		I	II		I	II		I	II		
E1 (Q1-Q7)	44	44.22 (3.61)	44.49 (3.82)	24	24.13 (4.66)	22.02 (5.55)	3.09	2.92 (0.70)	3.12 (0.89)	0	-0.00 (0.79)	-0.14 (1.13)	2.34	2.21 (0.95)	2.12 (1.70)	0	0.11 (1.14)	0.21 (2.87)	44.7	27.3
E2 (Q3-Q5)	50	50.04 (2.47)	50.27 (3.35)	15	15.41 (5.56)	15.05 (6.13)	2.6	2.34 (0.72)	2.65 (0.78)	-2.1	-1.84 (1.31)	-2.17 (1.09)	0	0.04 (2.12)	-0.01 (1.16)	3.2	2.93 (1.60)	3.21 (1.57)	81	49
E3 (Q6-Q7)	75	75.03 (4.62)	75.44 (5.03)	24	24.07 (4.64)	23.83 (4.94)	-3.7	-3.33 (0.69)	-3.70 (0.82)	0	-0.05 (0.80)	-0.29 (1.24)	0	-0.06 (0.85)	0.16 (1.27)	-1.9	-1.74 (1.28)	-2.20 (1.63)	58	32.7

^a ^Epi.: the paired QTLs (in the parenthesis) involved in epistatic effects; Pos. *i *and Pos. *j*: the positions of the QTLs measured by the distance (cM) between a QTL and its corresponding first marker on the same chromosome; *AA*: additive-additive effect; *AD*: additive-dominance effect; *DA*: dominance-additive effect; *DD*: dominance-dominance effect; Power: the percentage of the QTL pair that is identified correctly; Par. (parameter): the true parameter value in simulations; Est.: the estimate of parameter; *SD*: the standard deviation of the estimate from the true value that is listed in the parenthesis where applicable; E1, E2, and E3: three epistases; I (Model I) and II (Model II): model (1) (silkworm F_2 _model) and the normal chiasmate F_2 _model, respectively.

In simulations, three different strategies were employed to conduct a genome-wide search for QTLs. The first one used the proposed model (1) (the silkworm F_2 _model), called Model I hereafter, the second used the traditional chiasmate F_2 _model (i.e., all coefficients in model (1) are replaced with those determined according to the genetic structure of a normal chiasmate F_2 _population), called Model II hereafter, and the third used the reduced version of model (1) where all epistatic effects and interaction effects of epistasis with sex were excluded, called Model III hereafter. The above three strategies were used to analyze the same simulated data sets generated by the silkworm F_2 _model with QTL effects, QTL × sex interaction effects.

We first examined the performance of the newly proposed strategy (Model I) and the traditional strategy (Model II) in mapping for silkworm traits and demonstrated the potential bias and loss of power caused by Model II. These simulation results were summarized in Tables 2 and 3. As the role of epistasis in the genetic control of complex traits has been well recognized, the comparison between Model I and Model III could offer us insight into epistasis detection. These results are listed in Tables 2 and 5.

## Results

### Comparison of coefficients in models for the achiasmate and the normal chiasmate F_2 _populations

As shown by the formula of conditional probability in Table 1, we could see that each probability value in achiasmate model is approximately equal to a first order function of *r*_1_, *r*_2 _or *r*, while, each one in chiasmate model approximates to a second order function of recombination rate, suggesting that there should be considerable difference between the two models, which can potentially result in estimation bias and loss in accuracy.

**Figure 1:**
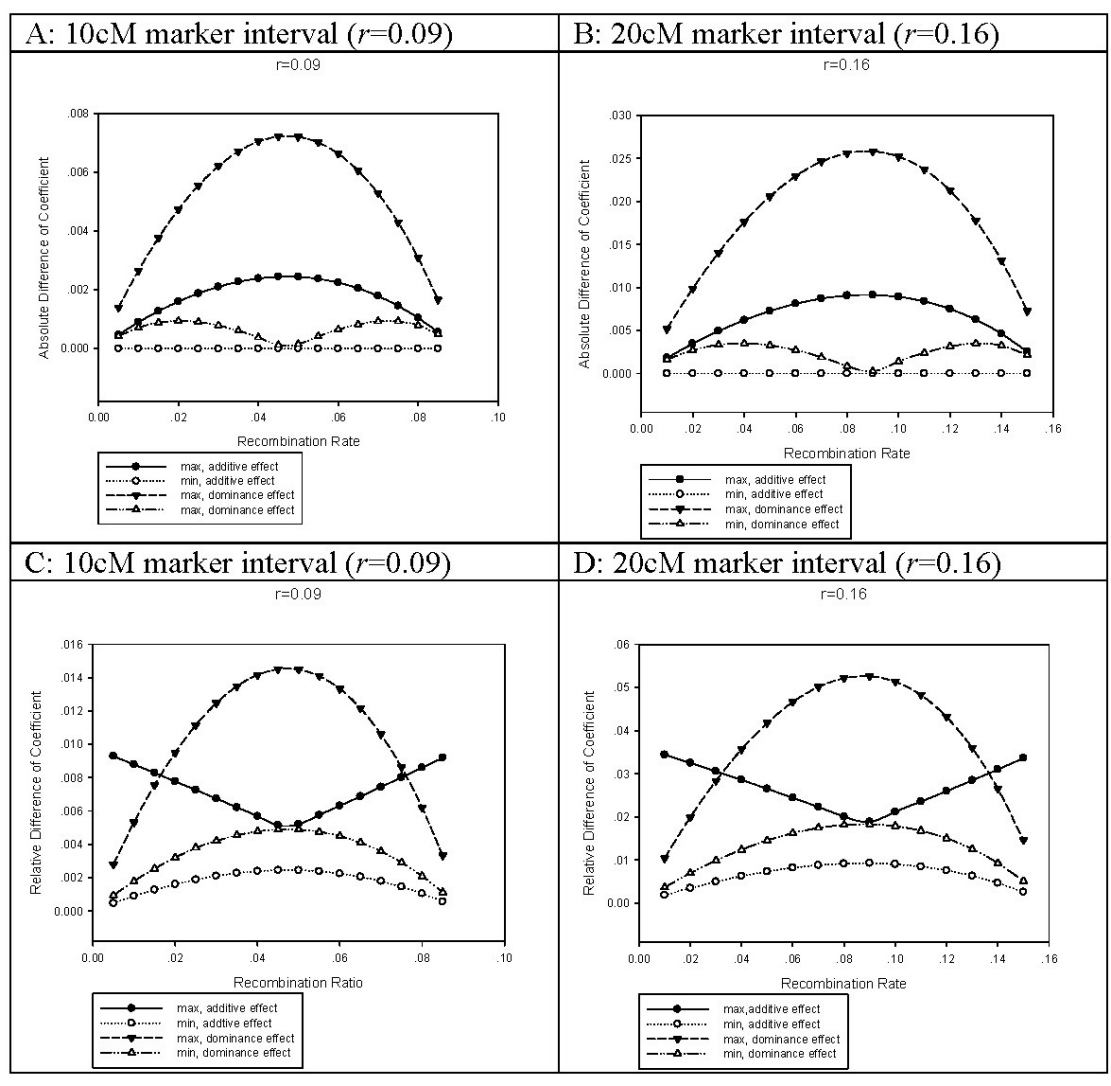
**Difference of coefficients in the achiamata and chiamata models**. x-axis stands for the recombination rate between QTL and left flanking marker and y-axis stands for the absolute value of coefficient difference or its percentage. Max curve is formed by the maximum absolute difference (or relative difference) for every different *r*_1_, while min curve by the minimum. Plot A and C were drawn at the marker interval of 10 cM (*r *= 0.09), and Plot B and D at the marker interval of 20 cM (*r *= 0.16).

The numerical examples could also illustrate this point. In the setting of *r *= 0.09, the absolute difference of coefficient ranged from 0 to 0.0025 for additive effect and from 0.0001 to 0.0050 for dominance effect (Figure 1A, respectively, while it did from 0 to 0.0090 for additive effect and from 0.0002 to 0.0170 for dominance effect under the setting of *r *= 0.16 (Figure 1B). The relative absolute difference varied in range of 0.0005 to 0.0093 for additive effect and in range of 0.0009 to 0.0145 for dominance effect when *r *= 0.09 (Figure 1C), while it did in range of 0.0018 to 0.0344 and in range of 0.0036 to 0.0526 when *r *= 0.16 (Figure 1D). It was also very clear that the maximum or minimum difference was reached when the QTL was at the middle of the marker interval.

According to the above comparison, it could be concluded that the traditional chiasmate *F*_2 _model would lead to a biased estimation when it was applied to mapping QTLs underlying silkworm traits and our proposed method would improve QTL mapping accuracy.

### Comparison between the models for the achiasmate silkworm and the normal chiasmate F_2 _populations

Model I and the traditional Model II were used to analyze the simulated data, and were compared for their abilities in estimating the position and genetic effects of QTLs. The results suggested that Model I had better estimation accuracy (relatively smaller bias and standard deviation) in QTL position and effects than Model II (Table 2). All bias of genetic main effects from Model I were less than 5% of the true values, whereas Model II sometimes gave a larger bias, e.g., the bias of the Q5 additive effect > 10% of the true value (Table 2). Model I had a considerably larger power to detect the five QTLs (ranging from 62.7 ~ 98.3) than Model II (ranging from 49.7 ~ 94.7) (Table 2), regardless of whether the QTL has interaction effects with sex (Q2, Q3, Q4) or not (Q1, Q5) (Table 5). For all QTLs detected in the 300 simulations, the false discovery rate of Model I is 6.17%, prominently smaller than that of Model II (9.56%) with a z-statistic value of -2.063 and a probability of 0.020 by Wilcoxon two-sample test. Model I also provided an estimation of QTL position closer to the true value and had smaller standard deviation (*SD*) than the model II did (Table 2).

We also compared the estimation accuracies of epistatic effects from Model I with those from Model II (Table 3). Although both the Model I and the Model II could estimate all epistatic effects reasonably well, Model I had relative smaller *SD *and greater power than Model II in detecting the three pairs of QTLs involved in epistatic interactions, wherein E1 stands for the interaction between one QTL with main effects and another without main effects, E2 stands for the interaction between both QTLs with main effects and E3 stands for the interaction between two QTLs without main effects. As for estimation of QTL position, Model I also outperformed Model II in accuracy.

### Comparison of the silkworm F_2 _models with and without epistasis

For the estimation of QTL positions, it could be found there was a slight difference in the estimated values between two models when the detected QTL has additive or dominance effects, or their interaction effects with sex, as well as in the corresponding *SD*s (Table 2). Two models had the same power in detecting QTL (Table 2), although Model I included more parameters of QTL than Model III. As for the paired QTLs with purely epistatic effects, they couldn't be identified by Model III, but could be detected by Model I. Such a result is expected, considering that (1) Model I and Model III used the same marker genotype information and quantitative trait values, (2) one dimensional scanning procedure did not include epistasis in Model III, and (3) the position of QTL was determined by the result of one dimensional scanning.

Although there was a small difference in position estimation, the estimates of additive and dominance effects were apparently different between two models (Table 2). Most of the estimated values in Model I were closer to the true values as compared with those in Model III. We could also see that, in Model I every estimate of additive or dominance effects had smaller *SD *than that in Model III, suggesting that Model I could provide more stable and more unbiased estimation (Table 2). As for QTL × sex interaction, although Model III could also give relatively accurate estimation, it was clear that the results of Model I were better than those of Model III, in terms of biasedness or *SD *(see Table 5).

### Prediction of QTL × sex interaction effects

Based on the QTL model proposed for silkworm F_2 _population in this article, we could obtain the unbiased prediction of random effects including additive-sex, dominance-sex and epistasis-sex interaction effects by the MCMC method. In the simulation studies, Model I provided not only the estimation of the genetic effects of QTLs including epistasis but also their interaction effects with sex (shown in Tables 4 and 5). For two kinds of QTLs involved in interaction with sex (Q2, Q3, Q4) or not (Q1, Q5), the predicted values of additive × sex and dominance × sex interaction effects were very close to the true realized values of random effects, and the corresponding *SD*s were acceptably low (Table 5). For the interaction between epistasis and sex (Table 4), all four types of interaction effects could be well predicted by Model I. Although slightly bigger bias and *SD*s were observed, compared with the results for additive or dominance × sex interaction effects, they were still acceptable.

**Table 4:** Estimation of epistasis-sex interaction effects with Model I ^a^

**Epi**.	*AAS1*		*AAS2*		*ADS1*		*ADS2*		*DAS1*		*DAS2*		*DDS1*		*DDS2*	
	
	**Par**.	Est.(*SD*)	**Par**.	Est.(*SD*)	**Par**.	Est.(*SD*)	**Par**.	Est.(*SD*)	**Par**.	Est.(*SD*)	**Par**.	Est.(*SD*)	**Par**.	Est.(*SD*)	**Par**.	Est.(*SD*)
E1	0	-0.05(0.39)	0	0.05(0.39)	1.7	1.25(0.95)	-2	-1.25(0.95)	0	0.05(0.44)	0	-0.05(0.44)	0	-0.00(0.66)	0	0.01(0.66)
E2	-1	-1.02(0.75)	1.4	1.04(0.75)	1.6	1.05(0.99)	-2	-1.05(0.99)	0	-0.02(0.43)	0	0.02(0.43)	0	-0.03(0.62)	0	0.03(0.62)
E3	0	-0.00(0.40)	0	0.00(0.40)	0	0.03(0.54)	0	-0.03(0.54)	1.7	1.39(1.00)	-2	-1.38(1.00)	1.8	1.12(1.27)	-2	-1.12(1.28)

^a ^*AASi *(*i *= 1,2): interaction effect between additive-additive epistasis and sex; *ADSi *(*i *= 1,2): interaction effect between additive-dominance epistasis and sex; *DASi *(i = 1,2): interaction effect between dominance-additive epistasis and sex; *DDSi *(i = 1,2): interaction effect between dominance-dominance epistasis and sex; Par.: the true parameter value in simulations; Est.: the estimate of parameter; *SD*: the standard deviation of the estimate from the true value that is listed in the parenthesis where applicable; Epi.: the paired QTLs involved in epistatic effects; E1, E2, and E3: three epistases.

**Table 5:** Estimation of QTL-sex interaction effects with Model I and Model III ^a^

QTL	*AS1*			*AS2*			*DS1*			*DS2*		
	
	**Par**.	Est.(*SD*)		**Par**.	Est.(*SD*)		**Par**.	Est.(*SD*)		**Par**.	Est.(*SD*)	
								
		I	III		I	III		I	III		I	III
Q1	0	0.02(0.28)	-0.01(0.29)	0	-0.02(0.28)	0.01(0.29)	0	-0.01(0.33)	-0.01(0.36)	0	0.00(0.33)	0.01(0.36)
Q2	-1.7	-1.53(0.42)	-1.52(0.48)	1.7	1.54(0.42)	1.53(0.48)	0	0.02(0.40)	0.04(0.38)	0	-0.01(0.39)	-0.03(0.38)
Q3	-1.6	-1.53(0.41)	-1.54(0.47)	1.6	1.54(0.41)	1.54(0.47)	-1.5	-1.28(0.60)	-1.23(0.74)	1.4	1.30(0.60)	1.19(0.71)
Q4	0	-0.02(0.22)	-0.03(0.26)	0	0.02(0.22)	0.03(0.26)	1.6	1.36(0.65)	1.30(0.75)	-1.6	-1.36(0.65)	-1.30(0.75)
Q5	0	0.02(0.22)	-0.01(0.27)	0	-0.02(0.22)	0.01(0.27)	0	0.01(0.29)	0.01(0.38)	0	-0.01(0.29)	-0.01(0.38)

^a ^*ASi *(*i *= 1,2): additive-sex interaction effect; *DSi *(*i *= 1,2): dominance-sex interaction effect; Par.: the true parameter value in simulations; Est.: the estimate of parameter; *SD*: the standard deviation of the estimate from the true value of parameter that is listed in the parenthesis where applicable; I and III: Model I and Model III, respectively.

## Discussion

Crossing-over is an important issue in organisms with meiosis, which can not only enrich phenotypic variation among individuals but also speed up the process of evolution. However, there are many exceptions such as Drosophila and silkworm, which are not only of great value as model organism for biology study but also important for agriculture. Drosophila and silkworm have a common characteristic in reproduction that crossing-over occurs only in one sex, while there is a difference that such a phenomenon occurs in female parent for silkworm and in male parent for Drosophila. The particular action in meiosis, also called achiasmata, needs to be considered in the process of gene mapping. In order to avoid the problem on the achiasmate and use available genetic model and software for a normal chiasmate mapping population, investigators have proposed to conduct QTL analysis based on BC population of silkworm. However, there are obvious disadvantages in this solution: recombination information and diversity of genotype in the BC population are not so rich as the F_2 _population for unraveling genetic architecture of complex traits where complex epistasis and gene × sex or environment interaction are involved. The existing studies on constructing linkage map or genetic mapping with F_2 _populations for silkworm [5,44] chose to simply neglect such a recombination difference between two sexes because of lack of appropriate analytical method. This potentially leads to bias and loss in precision as, in silkworm F_2 _population, every individual receives one gamete from female parent without crossing-over and another one generated by potential sister-chromatid exchange from male parent, resulting in a different population structure from the normal chiasmate F_2 _population. Thus, our proposed method that can accommodate achiasmata phenomenon and also effectively handle epistatic effects and the interaction effects of QTL and environment, represents a necessary addition to the current toolkit of QTL mapping.

Many of the widely used statistical methods and software, such as IM and CIM, do not include sex effects in the models because they are mainly designed for plants such as Arabidopsis thaliana and rice. But in animals, many quantitative traits are sex dependent and behave much differently between males and females, such as the cocoon traits of silkworm. Sex specific traits can be categorized into three types of inheritance: sex-limited, sex-influenced (also known as sex-controlled), and sex-linked; the former two of which are controlled by autosomal gene(s) and sex, in our term, in which there are sex and/or gene × sex interaction effects, and the latter one is caused by gene(s) carried on the sex chromosomes. It has been well documented that there are sex differences in terms of the presence/absence and locations of QTLs [45], as well as the interaction of QTL with sex [46-48]. Thus, for the purpose of improving analysis power, the sex effect and the QTL × sex interaction effect should be generally included into the analysis model as a covariate to eliminate the influence from sex. In our study, as in some literature [49], the sex effect is considered in the QTL model as a random effect for the purpose of background control. Simulation results showed the proposed method could improve both statistical power to detect a QTL and estimation accuracy for genetic effects of QTL and QTL × sex interaction. We like to point out that the sex and the sex related interaction effects can also be treated as fixed ones in our model when necessary.

The method presented here is mainly to detect QTLs on non-sex chromosomes. Sex chromosomes usually play a unique role in many biological processes and phenomena, including sex determination, epigenetic chromosome-wide regulation of gene expression [50]. Sex chromosomes have many different genetic features compared with autosomes and there is extra complexity in mapping of sex-linked genetically inherited traits. First, there are two categories of sex determination systems: heterogametic male (XY) and heterogametic female (ZW), and the heterogametic sex is hemizygous in which gene dosage effect or dosage compensation mechanism may occur. Second, sex chromosomes can show sex-biased transmission. Third, there may also exist random inactivation of the sex chromosome. Broman et al. [51] addressed that if the sex chromosome is treated like an autosome, a sex difference in the phenotype can lead to spurious linkage on the sex chromosome. Further, the number of degrees of freedom for the linkage test may be different for the sex chromosome than for autosomes, and so sex chromosome-specific significance threshold is required. Given the complexity of sex-linked inheritance, tailored mapping methods are needed to effectively hunt sex-linked genes. Thus, Broman et al. [51] proposed a method for mapping QTL on X chromosome in experimental crosses population. Zhang et al. [52] developed a family-based association test to detect QTLs on X-chromosome under consideration of the dosage effect due to female X chromosome inactivation. It is possible to extend the proposed method to mapping QTLs on the sex chromosomes.

The genetic variation in continuous traits is usually governed by a polygenic network system, composed of many genes with a small effect, and sometimes one or a few genes of large effect. Recently, intensive studies on quantitative variation have pointed to that epistasis is usually involved in genetic variation of quantitative traits. Strong interactions between QTLs have been observed in maize [53], soybean [54] and *Drosophila *[55]. In addition, QTLs with minor or no individual effect can also be involved in epistatic interaction [56]. More and more attention has been paid to molecular dissection of epistasis. Our proposed model includes not only the digenic epistatic effects, but also their interaction effects with sex. Therefore, this model can well tackle the complexity of quantitative trait in silkworm. Simulations revealed that the proposed method could present better estimation of QTL parameters no matter whether or not the epistasis and/or their interaction with sex exists.

Lastly but not least, it should be pointed out that only seven different genotypes of two QTL loci on the same chromosome can be generated in F_2 _population because of the female achiasmate of silkworm [2], while, in the full model, eight genetic effects of a pair of QTLs (two additive effects, two dominance effects and four epistatic effects) need to be estimated. Therefore, under this situation, the proposed method could not produce unbiased estimate of all eight fixed effects. An elaborately planned design is required to effectively detect epistases between interacting loci located on the same chromosome due to the insufficient number of segregating genotypes in an achiasmate F_2 _population. One alternative choice is excluding the higher-order genetic effects of QTL (additive-dominance, dominance-additive, dominance-dominance epistatic effects) from the model. However, for the case in which two QTLs are residing on two different chromosomes, there are still nine QTL genotypes segregated in F_2 _population of silkworm since the chromosome of female parent could be passed independently to its progeny. It explained why Model I could well estimate all epistatic effects in our simulation study. Furthermore, fortunately, the position of QTL could be estimated unbiasedly no matter whether the QTLs are distributed on the same chromosome or not, since the QTL position is distinguished based on the *F*-statistic measuring the total extra effects due to tested variables in the model which is not affected by the correlation between these variables.

## Conclusion

We have developed a genetic model for mapping QTL in silkworm F2 population which could analyze the additive effect, dominance effect, digenic epistatic effect and their interaction effects with sex, and correct the potential bias and precision loss in the current QTL mapping practice of silkworm, thus representing an important addition to the arsenal of QTL mapping tools.

## Authors' contributions

HMX and CSW developed methods, performed simulation analyses, interpreted results and drafted the manuscript. YTT, ZHZ and YFS helped performed simulation studies. XYL directed the development, design, simulations, interpretation of results, and drafted manuscript. All authors read and approved the manuscript.
